# Alcohol Screening and Brief Intervention in Police Custody Suites: Pilot Cluster Randomised Controlled Trial (AcCePT)

**DOI:** 10.1093/alcalc/agy039

**Published:** 2018-06-08

**Authors:** Michelle Addison, Ruth Mcgovern, Colin Angus, Frauke Becker, Alan Brennan, Heather Brown, Simon Coulton, Lisa Crowe, Eilish Gilvarry, Matthew Hickman, Denise Howel, Elaine Mccoll, Colin Muirhead, Dorothy Newbury-Birch, Muhammad Waqas, Eileen Kaner

**Affiliations:** 1 Institute of Health & Society, Newcastle University, Baddiley Clark Building, Richardson Road, Newcastle Upon Tyne, UK; 2 School of Health and Related Research, Health Economics and Decision Science, The University of Sheffield, Regent Court, 30 Regent Street, Sheffield, UK; 3 Nuffield Department of Population Health, University of Oxford, UK; 4 Centre for Health Services Studies, University of Kent, Canterbury, Kent, UK; 5 Newcastle & North Tyneside Addictions Service, Plummer Court, Carliol Place, Newcastle upon Tyne, UK; 6 School of Social and Community Medicine, University of Bristol, Canynge Hall, 39 Whatley Road, Bristol, UK; 7 School of Health and Social Care, Teesside University, Middlesbrough, UK; 8 Economics Division, Leeds University Business School, Leeds, UK

## Abstract

**Aims:**

There is a clear association between alcohol use and offending behaviour and significant police time is spent on alcohol-related incidents. This study aimed to test the feasibility of a trial of screening and brief intervention in police custody suites to reduce heavy drinking and re-offending behaviour.

**Short summary:**

We achieved target recruitment and high brief intervention delivery if this occurred immediately after screening. Low rates of return for counselling and retention at follow-up were challenges for a definitive trial. Conversely, high consent rates for access to police data suggested at least some outcomes could be measured remotely.

**Methods:**

A three-armed pilot Cluster Randomised Controlled Trial with an embedded qualitative interview-based process evaluation to explore acceptability issues in six police custody suites (north east and south west of the UK). Interventions included: 1. Screening only (Controls), 2. 10 min Brief Advice 3. Brief Advice plus 20 min of brief Counselling.

**Results:**

Of 3330 arrestees approached: 2228 were eligible for screening (67%) and 720 consented (32%); 386 (54%) scored 8+ on AUDIT; and 205 (53%) were enroled (79 controls, 65 brief advice and 61 brief counselling). Follow-up rates at 6 and 12 months were 29% and 26%, respectively. However, routinely collected re-offending data were obtained for 193 (94%) participants. Indices of deprivation data were calculated for 184 (90%) participants; 37.6% of these resided in the 20% most deprived areas of UK. Qualitative data showed that all arrestees reported awareness that participation was voluntary, that the trial was separate from police work, and the majority said trial procedures were acceptable.

**Conclusion:**

Despite hitting target recruitment and same-day brief intervention delivery, a future trial of alcohol screening and brief intervention in a police custody setting would only be feasible if routinely collected re-offending and health data were used for outcome measurement.

**Trial registration:**

ISRCTN number: 89291046.

## INTRODUCTION

An extensive body of international evidence demonstrates a link between alcohol consumption, risky behaviours and criminal activity (Miller *et al.*, 2006; Newbury-Birch *et al.*, 2009; Barton, 2011; Bouchery *et al.*, 2011; Kennedy *et al.*, 2012; Kinner *et al.*, 2015; Needham *et al.*, 2015; Orr *et al.*, 2015; de Andrade *et al.*, 2016). Alcohol-related crimes have been estimated to cost £11 billion per annum in the UK (Home Office, 2013) and between $73 and $84 billion in the USA (Miller *et al.*, 2006; Bouchery *et al.*, 2011). The offender population has a high prevalence of heavy drinking with between 64% and 84% of offenders reporting hazardous, harmful or dependent drinking (Newbury-Birch *et al.*, 2009; Brown *et al.*, 2010; Kinner *et al.*, 2015; Orr *et al.*, 2015). A quarter of police time is focused on dealing with alcohol-related crime in the UK (Palk *et al.*, 2007) with alcohol linked to half of all violent crimes (Flatley *et al.*, 2010; Birch *et al.*, 2015). Thus, police custody suites present a unique opportunity to intervene with heavy drinkers (Newbury-Birch *et al.*, 2009; Brown *et al.*, 2010; McCracken *et al.*, 2012; Orr *et al.*, 2015) and prevent harmful consequences for arrestees and crime victims (stein *et al*., 2010; Barton, 2011; Orr *et al.*, 2015; Newbury-Birch *et al.*, 2016).

Screening and brief alcohol interventions are effective at reducing heavy drinking, particularly in community-based health settings (Kaner *et al.*, 2017), and are being considered for use in the criminal justice context (Brown *et al.*, 2010; Blakeborough and Richardson, 2012; Coulton *et al.*, 2012; Graham *et al.*, 2012; Home Office, 2013). A longitudinal survey of 1325 adult prisoners in Australia, assessed the predictive validity of the Alcohol Use Disorders Identification Test (AUDIT) and found that pre-release AUDIT scores predicted hazardous drinking 6 months after release (Thomas *et al.*, 2014). As detention in police custody typically occurs relatively soon after an offence is committed it may provide a ‘teachable moment’ to link drinking behaviour with offending behaviour (Schmidt *et al.*, 2015). Alcohol screening can identify offenders who may benefit from targeted brief intervention (Brown *et al.*, 2010; Coulton *et al.*, 2012; Graham *et al.*, 2012; Home Office, 2013; McGovern *et al.*, 2018, under review). However, Orr et al. (2015) examined the feasibility of delivering brief alcohol interventions in a community justice setting where 42% of participants (*n* = 195) were hazardous/harmful drinkers and found that just 15% were followed up at 3 months; the low retention rate was ascribed to group transience and mistrust. Nevertheless, the English Home Office piloted alcohol arrest referral schemes to test whether brief interventions could reduce re-offending across 12 police forces between 2007 and 2010 (Blakeborough and Richardson, 2012). This scheme employed alcohol specialists to deliver brief interventions to arrestees with alcohol-related problems and refer to treatment services if required. This non-randomised evaluation showed statistically significant reductions in alcohol consumption at follow-up, but the comparison was only with retrospective controls. To date, most alcohol intervention studies based in criminal justice settings have been small, exploratory and/or non-randomised evaluations (Man et al., 2002; Brown *et al.*, 2010; Barton, 2011; Blakeborough and Richardson, 2012; Coulton *et al.*, 2012). To our knowledge, this is the first pilot randomised controlled trial aimed at assessing the feasibility and acceptability of a definitive evaluation of alcohol screening and brief intervention delivery in police custody suites (where arrestees are processed and detained).

## METHODS

The pilot trial protocol has been published previously (Birch *et al.*, 2015). The study was based on six custody suites across four police forces: three forces in the North East (Tyne and Wear, Durham, Cleveland) and one force South West of the UK (Bristol).

Detention Officers and/or Assessment and Intervention Referral Staff (AIRs) were cluster randomised with equal probability to one of the three trial arms using random permuted block randomisation. AIRs are specialist staff who identify detainees with alcohol-related problems, provide brief alcohol interventions, and refer them into alcohol treatment services. Randomisation was stratified by police custody suite and conducted independent from the research team. All staff received the same training in screening and brief advice procedures.

The arm to which staff were allocated was placed in a sealed opaque envelope, with a unique ID number. Neither the trial statistician nor trial staff delivering training were aware of the allocation prior to commencement of training.

## INCLUSION AND EXCLUSION CRITERIA

Custody suites, and staff, were eligible for inclusion in the cluster trial if they were within the specified regions. Eligible arrestees were aged 18 or over; alert and orientated; able to speak, read and write English; and have a fixed abode.

Exclusion criteria included serious mental health problem, being injured or grossly intoxicated (eligibility determined by staff once sober), currently seeking help for alcohol problems.

Eligible arrestees were given an information leaflet and received verbal communication from Detention Officers/AIRs about the purpose of the trial. The arrestee was asked to provide verbal consent for screening. Participants scoring 8+ on the Alcohol Use Disorders Identification Test (AUDIT, score range 0–40) (Babor and Higgins-Biddle, 2001) were enroled and asked to give written, informed consent, contact details and preferred mode of follow-up.

## INTERVENTIONS

All staff received the same training in screening and (if relevant) brief advice procedures by the research team. Competence was assessed through weekly targets and feedback, and booster training sessions were provided specifically to the north-east sites to improve screening rates.

The three (additive) trial arms were:

- Screening only (control group);
- 10 min of manualised brief structured advice delivered by detention officers/AIRs who carried out screening (intervention 1); and;
- 10 min of manualised brief structured advice followed by 20-min of manualised brief counselling delivered by trained alcohol counsellors (intervention 2). Brief counselling was intended to support a more in-depth understanding of alcohol use drivers and consequences including links with offending behaviour and impacts on other people. (Henry-Edwards *et al.*, 2003; Newbury-Birch et al., 2014; Birch *et al.*, 2015).

**Fig. 1.**
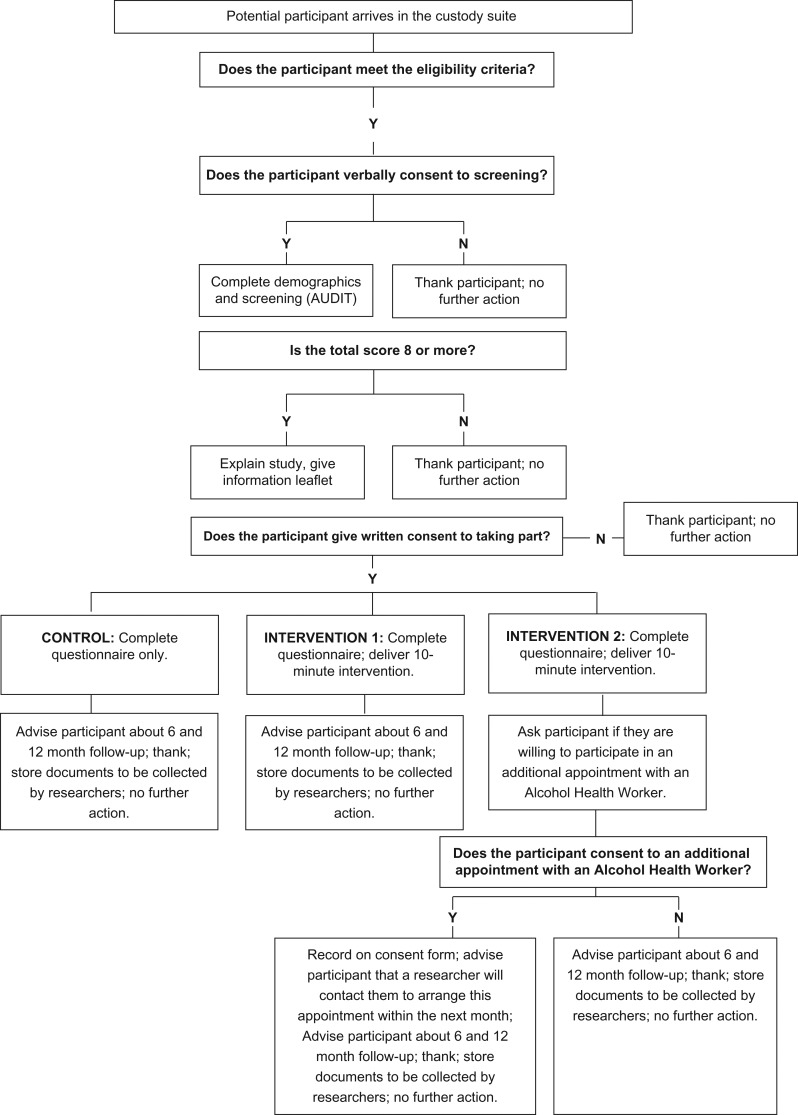
Study process.

In all North East sites, brief counselling was delivered by an alcohol counsellor within 1 month of initial input. In the South West, brief counselling was offered and delivered on the same day as randomisation by trained AIRs who had carried out the screening/brief advice. Fig. 1 provided details of the trial processes.

### Primary outcome measures

Key outcome measures for the pilot trial:

- Percentage of eligible participants enroled at baseline.
- Percentage of enroled participants followed up at 12 months.

**Fig. 2.**
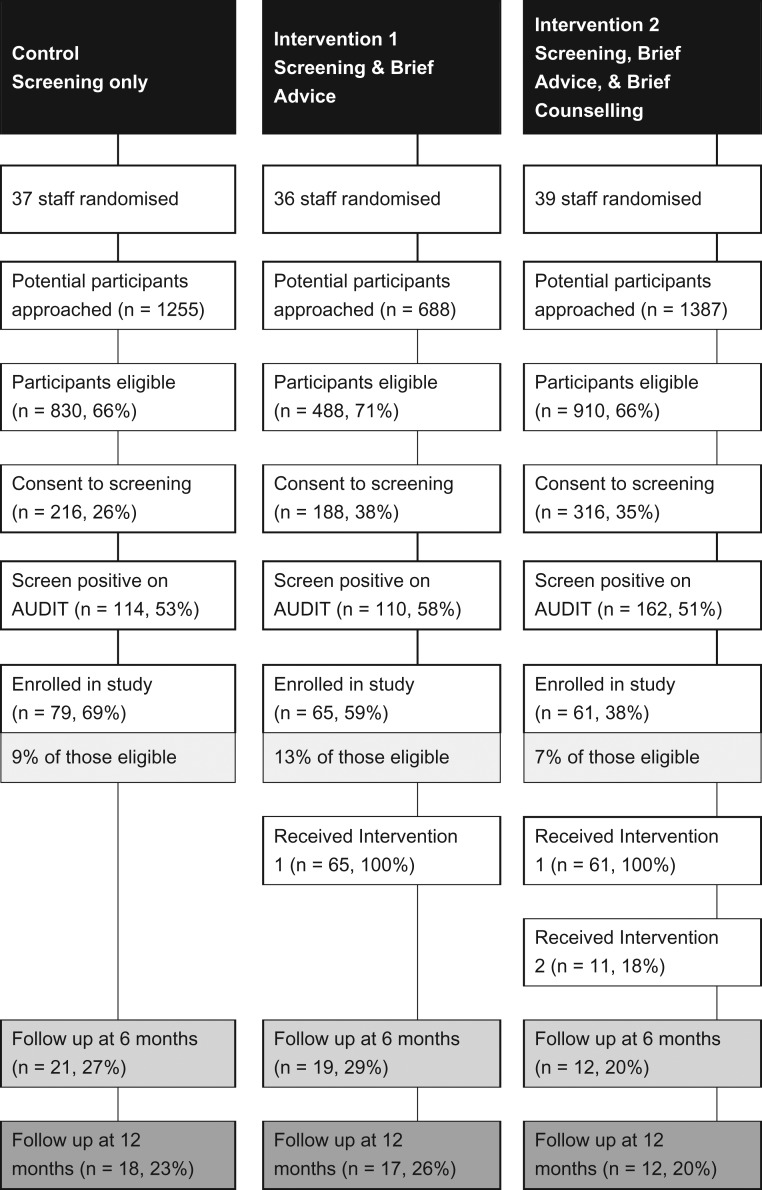
Trial consort diagram.

Due to uncertainty about the mobility and traceability of the study population, 6-month follow-up was carried out to re-check contact details and assess interim attrition. Fig. 2 reports the trial consort diagram.

### Secondary outcome measures

A number of tools were administered to assess response variability in these measures which include:

- Ten item AUDIT (score range 0–40): AUDIT score has been found to be responsive to change following alcohol intervention and successfully used as an outcome measure in a recent trial with offenders (Newbury-Birch *et al.*, 2014). AUDIT scores were categorised as 0–7 (low-risk drinking: for non-cases^1^ only); 8–15 (hazardous drinking); 16–19 (harmful drinking); 20–40 (probable dependent drinking) and unknown.
- The modified Readiness to Change Ruler assessed readiness to change drinking behaviour on a numerical scale of 0–10 (Birch *et al.*, 2015) and median score reported.
- EQ-5D-5L measured Health-Related Quality of Life (Janssen *et al*., 2013; Birch *et al.*, 2015; Mulhern *et al.*, 2018).
- Arrest data: permission was sought from participants at enrolment for linkage to police force arrest data. This was possible using the Criminal Record Number allocated to the reason for arrest, and a unique Serial Record Number. Number and type of arrest were sought for the 12 months before screening (including the current arrest) and the 12 months following intervention. These data were collected via data sharing protocols agreed with senior police staff in each force area.
- Indices of Multiple Deprivation (IMD) reported as quintiles of deprivation (see Table 1): 1 represented the most deprived areas (2017). Police force arrests data also contained arrestees’ contact details, including postcodes which were used to calculate IMDs.

**Table 1.** Demographic characteristics

Characteristics	Total cases *n = 205*	Control	Intervention 1 (BI)	Intervention 2 (BI & BCC)
		*n* = 79	*n* = 65	*n* = 61
	*n*/%	*n*/%	*n*/%	*n*/%
Age	32.47 m, (10.96 sd)	32.46 m, (10.85 sd)	32.3 m, (10.63 sd)	33.2 m, (9.3 sd)
Males	170 (82.9)	70 (89)	51 (78)	49 (80)
Females	35 (17.1)	9 (11)	14 (22)	12 (20)
Ethnicity				
White	193 (94.1)	76 (96)	60 (92)	57 (93)
*Status*				
Single	136 (66.3)	53 (67)	41 (63)	42 (69)
Married or living with partner	49 (24)	15 (19)	18 (28)	16 (26)
*Education*				
Did not finish school	72 (35.1)	28 (35)	18 (28)	26 (43)
GCSE education	87 (42.4)	32 (41.5)	30 (46)	25 (41)
Smokers	150 (73.2)	61 (77)	45 (69)	44 (72)
Employment				
Employed	70 (34.1)	30 (38)	20 (31)	20 (33)
Seeking work	62(30.2)	23 (29)	19 (29)	20 (33)
Disability and sickness	18 (37)	12 (15)	13 (20)	12 (20)
*IMD of residence*—*quintiles*				
1 (most deprived)	77 (37.6)	36 (45.6)	23 (35.4)	18 (29.5)
2	56 (27.3)	23 (29.1)	16 (24.6)	17 (27.9)
3	18 (8.8)	6 (7.6)	4 (6.2)	8 (13.1)
4	24 (11.7)	7 (8.9)	9 (13.8)	8 (13.1)
5 (least deprived)	9 (4.4)	1 (1.3)	5 (7.7)	3 (4.9)
Unknown	21 (10.2)	6 (7.6)	8 (12.3)	7 (11.5)

### Statistical and economic analyses

No formal hypotheses were tested. All outcome measures were reported descriptively at baseline and (where relevant) also at the 6- and 12-month follow-ups (Tables 2 and 3).

**Table 2.** Reasons for being in custody for cases and non-cases

	Cases		AUDIT positive—did not consent to trial (non-cases)		AUDIT negative—ineligible for trial (non-cases)	
Reason for arrest	*N*	*%*	*N*	*%*	*N*	*%*
Acquisitive (burglary/theft)	49	23.9	30	16.6	112	33.7
Violence	42	20.5	48	26.5	64	19.3
Drink-related	28	13.7	25	13.8	14	4.2
Other	23	11.2	12	6.6	27	8.1
Public order	17	8.3	20	11.0	30	9.0
Criminal damage	12	5.9	14	7.7	17	5.1
Unknown	12	5.9	6	3.3	7	2.1
Drug-related	11	5.4	10	5.5	28	8.4
Sexual offences	4	2.0	5	2.8	11	3.3
Administrative	4	2.0	9	5.0	10	3.0
Driving-related	2	1.0	2	1.1	7	2.1
Domestic violence	1	0.5	0	0.0	5	1.5
Total	205	100	181	100	332	100

Note: Non-cases are arrestees who met the eligibility criteria and provided verbal consent to screening but were not included in the trial, because either (i) they did not score positive on AUDIT or (ii) they did score positive on AUDIT but did not provide written consent.

**Table 3.** Unit costs

Sr. no.	Item	Cost	Source
1	Detention Officer	£24,955 per year (exc National Insurance)	http://www.payscale.com/research/UK/Job=Detention_Officer/Salary
2	Assessment and Intervention Referral Staff (AIRs)	£31,914 per year; £56/h	PSSRU 2015 Page no. 65
*Hospital services*			
Q.1	A and E department visit as a patient	£140.59	NHS reference costs 2014/15 (‘Total Outpatient Attendances’, Accident and Emergency, Service Code 180)
Q.2	Hospital stay cost	£400	https://data.gov.uk/data-request/nhs-hospital-stay
Q.3	Hospital admission, no overnight stay	£720.78	NHS reference costs 2014/15 (‘Index’, Day Case, DC)
Q.4	Outpatient appointment cost	£134.22	NHS reference costs 2014/15 (‘Index’, Outpatient Procedures, OPROC)
*General practice service*			
Q.1	Doctor visit at GP practice	£44, £65 (depending on duration); please create two variables for both durations	PSSRU 2015 Page no. 177
Q.2	Doctor visit at home	use same rate as above for now	PSSRU 2015 Page no. 177
Q.3	Nurse visit at GP practice	£36 (£43 inc qualit) per hour; £47 (£56) per hour of face-to-face contact	PSSRU 2015 Page no. 174
Q.4	Nurse visit at home	£36 (£43) per hour; £47 (£56) per hour of face-to-face contact	PSSRU 2015 Page no. 174 No information given on average mileage covered per visit
Q.5	Prescription cost	£23.30	PSSRU 2015; Page no. 177 (Although, Alcohol-related prescriptions are not mentioned)
*Social and care services*			
Q.1	Visited by a social worker at home	£40 (£57) per hour; £55 (£79) per hour of client-related work	PSSRU 2015 Page no. 188 No information given on average mileage covered per visit
Q.2	Visited a social worker at their office	£40 (£57) per hour; £55 (£79) per hour of client-related work	PSSRU 2015 Page no. 188
Q.3	Visited by a (home) care worker or advisor	Face-to-face: £24 per hour weekday	PSSRU 2015 Page no. 192 No information given on average mileage covered per visit
Q.4	Visited a (home) care worker at their office	Face-to-face: £24 per hour weekday	PSSRU 2015 Page no. 192
*Criminal justice resources*			
Q.1	Been arrested or cautioned	£285 (detained) £593 (arrest with no further action simple caution)	http://gve.withanedge.co.uk/valuations/arrest-(and-detained)-(cost-to-police)/neweconomymanchester.com/media/1446/3316-150327-unit-cost-database-v1-4.xlsx
Q.2	Magistrate’s court appearance cost	Cost will depend upon type of proceeding	http://www.cps.gov.uk/legal/a_to_c/costs/annex_1_-_scales_of_cost/
Q.3	Crown court appearance cost	Cost will depend upon type of proceeding	http://www.cps.gov.uk/legal/a_to_c/costs/annex_1_-_scales_of_cost/
Q.4	Day spent in prison	£33,785 (per year) in year 2013/2014, inflated by 1.4% to 2014/2015 price according to GDP deflator (PSSRU 2015, p. 241): £34,258 (2014/2015)	neweconomymanchester.com/media/1446/3316-150327-unit-cost-database-v1-4.xlsx

The economic evaluation tested the feasibility of proposed methods for a definitive trial. Data collection tools for engagement with health, social and criminal justice services as well as health-related quality of life information were assessed by means of the proportion of missing data on questionnaires (including service use and EQ-5D) (see Table 4).

**Table 4.** Health-related quality of life (HRQoL): EQ-5D-5L

	Control						Intervention 1						Intervention 2					
	Baseline		6 Months		12 Months		Baseline		6 Months		12 Months		Baseline		6 Months		12 Months	
	*n*	%	*n*	%	*n*	%	*n*	%	*n*	%	*n*	%	*n*	%	*n*	%	*n*	%
	79		21		18		65		19		17		61		12		12	
																		
Mobility																		
I have no problems walking about	67	85	19	90	14	78	50	77	15	79	15	88	51	84	8	67	9	75
I have slight problems in walking about	4	5	1	5	1	6	9	14	1	5	1	6	4	7	2	17	3	25
I have moderate problems in walking about	2	3	0	0	2	11	1	2	3	16	1	6	3	5	2	17	0	0
I have severe problems in walking about	2	3	1	5	1	6	1	2	0	0	0	0	2	3	0	0	0	0
I am unable to walk about	0	0	0	0	0	0	0	0	0	0	0	0	0	0	0	0	0	0
Missing	4	5	0	0	0	0	4	6	0	0	0	0	1	2	0	0	0	0
*Self-care*																		
I have no problems washing or dressing myself	72	91	19	90	16	89	57	88	15	79	14	82	57	93	11	92	11	92
I have slight problems washing or dressing myself	2	3	1	5	0	0	2	3	3	16	1	6	1	2	0	0	0	0
I have moderate problems washing or dressing myself	1	1	1	5	2	11	0	0	0	0	1	6	2	3	0	0	1	8
I have severe problems washing or dressing myself	0	0	0	0	0	0	1	2	1	5	0	0	1	2	1	8	0	0
I am unable to wash or dress myself	0	0	0	0	0	0	0	0	0	0	1	6	0	0	0	0	0	0
Missing	4	5	0	0	0	0	5	8	0	0	0	0	0	0	0	0	0	0
*Usual activities*																		
I have no problems doing my usual activities	63	80	19	90	14	78	50	77	14	74	13	76	48	79	9	75	7	58
I have slight problems doing my usual activities	5	6	1	5	1	6	9	14	1	5	1	6	5	8	1	8	2	17
I have moderate problems doing my usual activities	3	4	0	0	2	11	1	2	1	5	1	6	3	5	2	17	3	25
I have severe problems doing my usual activities	3	4	0	0	1	6	1	2	2	11	2	12	4	7	0	0	0	0
I am unable to perform my usual activities	1	1	0	0	0	0	1	2	1	5	0	0	1	2	0	0	0	0
Missing	4	5	1	5	0	0	3	5	0	0	0	0	0	0	0	0	0	0
*Pain/discomfort*																		
I have no pain or discomfort	51	65	18	86	14	78	39	60	14	74	15	88	43	70	8	67	8	67
I have slight pain or discomfort	10	13	2	10	1	6	10	15	0	0	1	6	6	10	0	0	1	8
I have moderate pain or discomfort	10	13	0	0	2	11	11	17	5	26	0	0	8	13	2	17	2	17
I have severe pain or discomfort	3	4	0	0	1	6	2	3	0	0	1	6	2	3	2	17	1	8
I have extreme pain or discomfort	1	1	0	0	0	0	0	0	0	0	0	0	2	3	0	0	0	0
Missing	4	5	1	5	0	0	3	5	0	0	0	0	0	0	0	0	0	0
*Anxiety/depression*																		
I am not anxious or depressed	32	41	12	57	8	44	18	28	10	53	9	53	18	30	5	42	8	67
I am slightly anxious or depressed	14	18	4	19	5	28	15	23	2	11	2	12	12	20	3	25	1	8
I am moderately anxious or depressed	18	23	2	10	1	6	10	15	2	11	1	6	15	25	1	8	1	8
I am severely anxious or depressed	3	4	2	10	3	17	14	22	2	11	1	6	11	18	2	17	1	8
I am extremely anxious or depressed	8	10	0	0	1	6	3	5	3	16	4	24	5	8	1	8	1	8
Missing	4	5	1	5	0	0	5	8	0	0	0	0	0	0	0	0	0	0

Resource data linked to staff time inputs (training, screening or intervention delivery) was collected, but not systematically because of time pressures on staff within a busy custody suite environment.

### Qualitative process evaluation

Qualitative interview work examining the feasibility and acceptability of the trial was undertaken with purposive samples of staff and arrestees following the 12-month follow-up. Staff findings are reported in detail separately (McGovern *et al.*, 2018, under review). Arrestees were recruited on the basis of being successfully contactable at follow-up and willing to participate in a subsequent interview. All interviews were conducted using a semi-structured topic guide which focused on trial experience and acceptability. Community-based arrestees were interviewed by telephone; a small number of arrestees (*n* = 7) were interviewed face-to-face in prison. The majority of interviews were audio-recorded and transcribed verbatim. We were not permitted to take audio-recording equipment into prisons and so these data were recorded via written notes. Anonymised transcribed narrative accounts were used to enable thematic analysis of key issues for participants. These were coded and analysed by two researchers.

### Success criteria

A formal power calculation was not required in this pilot trial (Birch *et al.*, 2015). A minimum number of 30 participants per study arm (90 in total) at 12 months was recommended to estimate a parameter for a definitive trial (Lancaster *et al.*, 2004). *A priori* success criteria were to recruit and deliver interventions to 60 arrestees per condition and follow-up 50% of total enroled participants at 12 months (Lancaster *et al.*, 2004; Birch *et al.*, 2015). The follow-up rate was agreed in advance with funders due to the transient nature of arrestees. We assessed item completion rates for study outcomes, including relevant economic data. Acceptability was determined via an interpretive assessment of qualitative interview work with detention staff (McGovern *et al.*, 2018, under review) and arrestees.

Ethical approval was granted by Newcastle University Ethics Committee (reference number 00754/2014).

## RESULTS

Of 3330 arrestees approached, 2228 (67%) met the eligibility criteria, 720 (32%) provided verbal consent for screening with 386 (54% of those consenting) scoring 8 or more on the AUDIT. Subsequently, 205 arrestees (53%) provided written consent to be enroled in the trial.

Staff varied in the number of participants they enroled: 112 custody officers were randomly allocated to a trial arm and only 47 recruited any participants.

The mean number of arrestees screened by each staff member was 44 (range 1–325).

### Primary outcomes

In total, 79 arrestees were recruited into the control condition (screening only), 65 into Intervention 1 and 61 into Intervention 2. Brief advice was delivered to all arrestees (in Intervention 1 and Intervention 2) but only 18% of arrestees (*n* = 11) received brief counselling (intervention 2) primarily on site delivered immediately after screening/brief advice by AIRs.

Table 1 describes the demographic characteristics of the participants. The majority of the sample were white (94%), male (83%), median age of 31 (IQR 24–40), and educated to GCSE standard (42%) or less (35%); 73% were current smokers and 30% were unemployed. The mean AUDIT score was 22 (SD 10) and the median was 20 (IQR 13–30).

In terms of risk status, 34% were hazardous drinkers, 16% were harmful and 50% were potentially dependent drinkers. Just 20% of arrestees reported that they had ‘never thought about changing their drinking’ based on ‘Readiness to Change’ scores. Finally 65% of enroled arrestees lived in the two most deprived area quintiles in the UK IMD (2015);.

### Follow-ups

Follow-up rates were 29% at 6 months and 26% at 12 months; contact by telephone was most successful (61% of those successfully followed up at 6 months and 60% of those at 12 months). An assessment of follow-up methods at 12 months indicated that 38% of cases did not reply to two letters that they were sent, 13% did not answer the phone when called, and 16% had invalid contact details at 6 months.

### Reasons for custody

For trial participants, the most common reasons for being in custody were the violent crime (20% compared with 27% for non-cases) or acquisitive offences (24% compared with 17% for non-participants) (Table 2).

### Data linkage

**Fig. 3.**
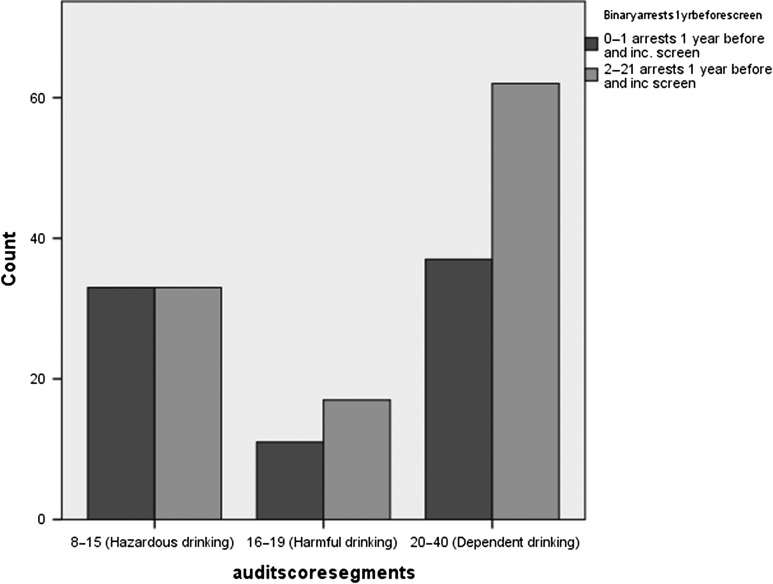
Numbers of arrests (0–1) or (2–21) amongst detainees enroled as cases in the 12 months before the study, by AUDIT category.

Permission was given by 94% (*n* = 193) of arrestees at baseline for linkage to police force data, and we obtained arrest/re-arrest data for 99% (*n* = 192) of these individuals (93% of cases in the trial). Arrest data values (see Fig. 3) ranged from 1 to 21 arrests in the year before the trial and 0–19 re-arrests in the 12 months following the intervention (before: median 2, IQR 1–4; after: median 0, IQR 0–2; and by trial arm/drinking category, Table 5).

**Table 5.** Number of arrests amongst cases in the 12 months before/after the trial

AUDIT category	Median *N* (IQR)	Median *N* (IQR)
	Before	After
8–15 (Hazardous drinking)	1 (1–3)	0 (0–2)
16–19 (Harmful drinking)	2 (1–3)	0 (0–2)
20–40 (Dependent drinking)	2 (1–5)	1 (0–5)
All	2 (1–4)	0 (0–2)
*Trial ARM*		
Control	1.5 (1–4)	1 (0–2)
Intervention 1	2 (1–4)	0 (0–2)
Intervention 2	2 (1–6)	0.5 (0–5)
All	2 (1–4)	0 (0–2)

### Economic evaluation

At 12 months follow-up, there was over 90% completion of all economic measures and no differences between the three trial arms (Table 4). Thus, questionnaires used to collect data appeared to be feasible for a full trial. Pilot trial data on costs associated with the delivery of the intervention were not sufficiently complete to provide a robust estimate of cost, but could be used to inform the design of a full trial and provide some information on the range of costs associated with each intervention. Data for the unit costs of resource use were collected from government sources wherever possible (Table 3).

While the number of participants available for either follow-up point was much lower than at baseline, responses to the EQ-5D-5L questionnaire were almost complete among participants who remained on the trial during the follow-up period, with a maximum of 8% of information missing.

### Qualitative findings

Interviews were conducted with 22 male arrestees (7 in prison) (*n* = 10 control, *n* = 9 intervention 1, *n* = 3 intervention 2).

Trial processes were generally well-received by many arrestees: ‘*I thought if I can give any help that might make people understand certain things and situations that maybe I have been through or whatever it might help’ (male, intervention 1).* Most also reported finding trial processes acceptable, *‘I didn’t feel any pressure to take part’ (male, intervention 1). However, o*nly arrestees who consented to the trial were interviewed, so their views may not be typical. There was clearly more reticence about being re-contacted at follow-up: ‘*I wouldn’t answer the phone if I was out of prison. I only said yes cos it’s boring and gives me someone to talk to’. (male, intervention 1, unrecorded).* Nevertheless, this view was not shared by all arrestees: *‘I’ve got no problem with you ringing me again’. (male, control, unrecorded).*

Arrestees’ motivation to participate varied from specific interest to a wish to alleviate boredom, but we found no evidence of coercion: *‘He actually came to the cell and said to us, ‘You can either stop in here for ten minutes or you can come out with me and fill this questionnaire out.’ I said, ‘Right, I’m coming out.’ (male, intervention 1)*. Arrestees demonstrated understanding about voluntary consent procedures (including access to routinely collected arrest data), ‘*I knew it was voluntary, yes’ (female, Control*) and ‘*I knew it was separate from the police and it was a university study’ (male, control, unrecorded).*

Finally, some arrestees reported that follow-up activity made them think about their drinking behaviour: *‘It was that odd call every few months, ‘Just seeing how you’re doing, how’s your drinking and stuff,’ and answering the same questions. It made me think about it more every time they did call.’* (male, intervention 1).

## DISCUSSION

We successfully recruited to target in all three trial arms and staff delivered screening and brief advice to 100% cases. However, only a third of eligible arrestees provided consent to be screened (Fig. 2). In addition, around half of trained staff did not recruit any arrestees into the trial. These challenges to recruitment could be because arrestees did not want any delays in being released from custody and because some staff felt too busy. It may be possible to improve arrestee consent rates in a future study by ensuring that screening and brief intervention occurred consistently at an earlier point in the detention process. Differences in staff views about role legitimacy are explored in a linked paper (McGovern *et al.*, 2018, under review). Only 18% of relevant participants received brief counselling (intervention 2). When the additional counselling was taken up, it was predominantly when input was offered on the same day as screening and brief advice. Other brief alcohol intervention studies have reported a significant drop-out of trial participants when counselling was offered on a subsequent occasion in primary care (Kaner, 2012), emergency care (Drummond *et al.*, 2014) and in an offender management context (Newbury-Birch *et al.*, 2014). Thus, immediate intervention would be necessary if a future trial took place.

Retention of arrestees at follow-up was challenging and just 26% of cases were re-contacted at 12 months. The similarity of the follow-up rates at 6 and 12 months suggested that there was no meaningful difference between them. Loss to follow-up was mainly due to participants moving address, changing their (mobile) telephone numbers or erroneous contact details (70% sample). Seven participants were in prison when re-contacted at follow-up, and one was deceased (reported by a family member). We were not able to offer financial incentives to encourage participation in this pilot trial as senior police staff were unhappy with this approach. Some suggestions were made about alternative forms of incentive such as phone top-up, vouchers or a certificate of participation in a research study.

Routinely measured data were available for most participants and the majority of participants in the trial (94%) gave permission for their police data to be accessed. These data provided rich information about numbers of arrests and offences. Contact details for participants were also checked as these are recorded at each arrest point. During the interview-based work, arrestees were positive about giving consent for health data to also be accessed and linked to police records. Linking up health and arrest data was also viewed as being acceptable in our public, participant and practitioners involvement work. With the correct governance approvals and consent processes, we are optimistic about future linkage to NHS data via GP/hospital records. Indeed, we were able to agree data sharing protocols regarding access to police data with all the forces in this study. Thus, use of routinely recorded and linked data could be a viable way of collecting post-intervention outcome data in a future trial. There were some issues with the collection of intervention costs for economic analysis, although we believe these could be overcome in a full trial with improved staff training. These data would allow a range of budget impact analyses to be undertaken. Although retention rates were low, follow-up EQ-5D-5L data were sufficiently complete to allow for a full cost-utility-analysis in a full trial.

Qualitative interview work indicated that trial processes seemed to be broadly acceptable to arrestees. In some instances, the follow-up process with arrestees indicated some potential screening and assessment reactivity (Kypri *et al.*, 2016). Most arrestees discussed the study intervention and procedures positively. Data relating to staff views are reported elsewhere (McGovern *et al.*, 2018, under review) and broadly positive, although views varied on which staff role was best suited to alcohol intervention work.

The clear need for alcohol intervention in police custody suites was confirmed by finding that 54% of screened arrestees were identified as having alcohol-related risk or harm; this was nearly twice the rate in the general population (Brown, 2016). However, half of these individuals reported AUDIT scores that were indicative of probable alcohol dependence (AUDIT score 20+) and likely to require further assessment, and potentially specialist care. These results are in line with other work in police settings (Newbury-Birch *et al.*, 2016). Nevertheless, this study found that arrestees were unlikely to return for a further appointment which presents challenges for the provision of more intensive treatment such as stepped care. In addition, a large proportion of study participants lived in areas of high social deprivation and were likely to experience multiple social disadvantages. Consequently, it seems important not to miss the opportunity to provide at least some positive support to help to address alcohol-related problems. Aside from the arrestees’ own levels of health risk and negative social harm due to being detained in the criminal justice system, the two most common reasons for the arrests in this study were violent and acquisitive offences which typically impact on other people. Thus, intervention with heavily drinking offenders may prevent adverse consequences for them, as well as reducing significant impacts on wider society linked to frequent re-offending behaviour.

## CONCLUSIONS

Taking all the outcomes together, we have mixed findings regarding the feasibility of a definitive trial of screening and brief alcohol interventions in a police custody suite context. Thus, we have an ‘amber status’ according to accepted criteria for progressing from pilot to definitive trials (Bugge *et al.*, 2013; Charlesworth *et al.*, 2013); ‘green’ indicates unequivocally supporting evidence and ‘red’ unequivocal evidence that future work is not feasible. Many aspects of the trial seemed acceptable and feasible including: positive site enrolment; achieving target participant recruitment; successful delivery of screening and of brief alcohol intervention, as long as this occurred on the same day as screening; and the reported acceptability of study procedures. Thus, if a future trial occurred, a two-armed trial (screening versus brief intervention) would be most efficient and any alcohol intervention content would need to be delivered on the same day as screening. However, whether the precise intervention content should be brief advice (intervention 1) or brief counselling (intervention 2) would need to be considered further. There is an accumulation of evidence which shows that brief counselling does not add significant additional benefit over simpler and shorter forms of brief alcohol intervention (Kaner *et al.*, 2017). Counselling also requires more skill, training and time than delivering structured advice. However, given the relatively high levels of alcohol-related risk in our study group and the context of frequent re-offending behaviour, more in-depth intervention may be required. A decision about the precise intervention content would require discussion with Custody Chief Inspectors about staff availability, skillsets and time available for alcohol intervention work (Scantlebury *et al.*, 2017b). It would be important to further explore arrestees’ views about their level of need and whether simpler or more in-depth interventions would be preferred (Scantlebury *et al.*, 2017a).

The most significant barrier to a future brief intervention trial based in a policing context is the low retention rates for arrestees, despite the fact that these were higher than reported in other recent similar work (Orr *et al.*, 2015; Scantlebury *et al.*, 2017a, 2017b). We did not achieve our target retention rate (50%) based on ‘in-person’ follow-up. However, we did achieve very high rates of consent for routinely recorded police data to be accessed, which provided an opportunity to accurately measure key criminal justice outcomes such as re-arrest rates. The arrestees who agreed to be interviewed were positive when asked about their future willingness to provide health system details (such as their name, date of birth and GP) and to have these data linked with police information, for research purposes. Consequently, a future trial would be feasible if intervention outcomes were measured via routinely collected criminal justice and health data rather than alcohol consumption (Johnson *et al.*, 2018). Indeed, although drinking behaviours are the most commonly reported outcome measures in brief alcohol intervention trials, these have been criticised as prone to bias due to socially desirable responding (Kypri *et al.*, 2016; McCambridge and Saitz, 2017). Consequently, objective health status or service use measures would have the advantage of reducing bias due to self-reported behaviour, however, they may be susceptible to recording and coding inaccuracy. Nevertheless, data-driven problems should be evenly distributed across trial arms in a randomised design and could help overcome challenges due to differential attrition reported in some alcohol intervention studies.

## CLINICAL TRIAL REGISTRATION

The outcomes of this ACCEPT Trial are linked to the qualitative findings regarding staff role security and therapeutic commitment; ISRCTN number: 89291046. Project start date: 01 April 2014; end date: 30 September 2016. After enrolment into study: 6-month follow-up period (18 December 2014—22 January 2016); 12-month follow-up period (24 July 2015—27 July 2016).

## GOVERNANCE AND ETHICS

The study sponsor was Newcastle University and ethics approval was granted by the Research Ethics Committee, Faculty of Medical Sciences at the same university (00754/2014). In addition to the published protocol, we compiled a data management plan which specified how all data were coded, anonymised and archived. The trial was overseen by the Newcastle Clinical Trials Unit.

## DISCLAIMER

The views expressed are those of the authors and not necessarily those of the NIHR SPHR.

## RELATIONSHIP STATEMENT

The NIHR School for Public Health Research is a partnership between the Universities of Sheffield, Bristol, Exeter, Cambridge, UCL; The London School for Hygiene and Tropical Medicine; the LiLaC collaboration between the Universities of Liverpool and Lancaster and Fuse; The Centre for Translational Research in Public Health, a collaboration between Newcastle, Durham, Northumbria, Sunderland and Teesside Universities. The study was led by Fuse investigators at the University of Newcastle upon Tyne, UK.
